# Efficacy and safety of traditional Chinese medicine decoction in the treatment of adolescent myopia: A protocol for systematic review and network meta-analysis

**DOI:** 10.1097/MD.0000000000028733

**Published:** 2022-02-11

**Authors:** Xiurong Tian, Zhongli Sun, Yonghua Li, Xianglin Jiang, Xingying Li, Penglong Yu

**Affiliations:** Chongqing Three Gorges Medical College, Chongqing, China.

**Keywords:** adolescent, myopia, network meta-analysis, protocol, systematic review, traditional Chinese medicine

## Abstract

**Background:**

Adolescent myopia has become a major public health problem in Asian countries and even the world. Due to its unstable prognosis and numerous complications, it has caused serious social and economic burden. As a common treatment in Asia, Chinese medicine has been shown to be effective in controlling the development of myopia, but its evidence-based medical evidence is not sufficient. Therefore, the purpose of this study is to evaluate the efficacy and safety of traditional Chinese medicine (TCM) in the treatment of adolescent myopia through network meta-analysis, and to provide evidence for clinical and scientific research.

**Methods:**

We searched seven databases for randomized controlled trials of TCM decoction for adolescent myopia, including PubMed, the Cochrane Library, EMbase, China National Knowledge Infrastructure, China Biological Medicine, Chinese Scientific Journals Database, and wan-fang databases, from the date of the establishment of each database to January 31, 2022. The network meta-analysis will be implemented through Aggregate Data Drug Information System 1.16.8 and Stata 13.0 software. Primary outcomes include distant vision, intraocular pressure, and diopter. Mean differences or odds ratios will be used for statistical analysis. We will ensure the reliability of the results through node-split model and heterogeneity analysis. In addition, the Cochrane Collaboration's tool and Grading of Recommendations Assessment, Development and Evaluation system will be used for the methodological quality and the evidence quality.

**Results:**

This study will provide reliable evidence for the clinical selection of TCM decoction in the treatment of adolescent myopia.

**Conclusion:**

The results of this study will evaluate the efficacy and safety of TCM decoction in the treatment of adolescent myopia, and provide decision-making references for future clinical and scientific research.

**Ethics and dissemination:**

This study did not require ethical approval. We will disseminate our findings by publishing results in a peer-reviewed journal.

**OSF registration number:**

DOI 10.17605/OSF.IO/VXQUP.

## 1 Introduction

Currently, myopia has become a major global public health problem in Asian countries.^[1]^ Around the world, 475.8 million people suffer from myopia by 2050.^[2]^ Myopia is also one of the main causes of visual impairment and has caused serious social and economic burden.^[3]^ Now, in China, the number of myopia patients remains high and continues to increase.^[4]^ Complications caused by excessive axial elongation caused by myopia, such as myopia macular, glaucoma, choroidal neovascularization, acquer cracks, etc., seriously affect the prognosis of myopia, which can lead to irreversible visual damage and heavy economy burden.^[5,6]^ In fact, in China, myopia macular degeneration is one of the main causes of low vision in adults and the second leading cause of blindness.^[7,8]^

Numerous studies have shown that low-dose (0.01%) atropine is effective in reducing the increase in myopia in adolescents, and topical atropine drops have been used for 150 years to relieve myopia.^[9–11]^ However, long-term use can cause photosensitivity and rebound effects after drug withdrawal due to concentration-dependent side effects, so higher concentrations of atropine drops are still not widely used.^[12]^

At present, a large number of classic Chinese medicine decoctions have been used in clinical treatment of adolescent myopia, and they have been made into different dosage forms at the same time, with good effects.^[13]^ However, there are many types of traditional Chinese medicine (TCM) decoctions in clinical application, and there is no interactive comparison between them. Therefore, the purpose of this study is to evaluate the efficacy and safety of different TCM decoctions in the treatment of adolescent myopia through network meta-analysis (NMA), and to provide reference and evidence for clinical application.

## 2 Methods

### 2.1 Protocol and registration

The NMA protocol has been registered on the Open Science Framework platform (https://osf.io/vxqup), registration number: DOI 10.17605/OSF.IO/VXQUP. This protocol follows the Preferred Reporting Items for Systematic Reviews and Meta-Analyses Protocols guidelines.^[14]^

### 2.2 Ethics

Since personal information of subjects is not required to be collected in this study, ethical permission is not applicable. In addition, adolescent subjects and their family members will sign informed consent during the study.

### 2.3 Eligibility criteria

The review will be conducted through the PICOS principle, including Participant (P), Intervention (I), Comparator (C), Outcome (O), and Study Design (S).

#### 2.3.1 Type of participant

All participants included in this study will meet the following criteria:

(a) be 18 years of age or younger;
(b) in this study, myopia will be defined as: equivalent spherical lens ≤-1.00 diopter after dilated eye examination;^[15]^
(c) not accompanied by any organic diseases of organs or eye diseases;
(d) those who have participated in any surgery or treatment related to vision correction will not be included.

#### 2.3.2 Type of interventions and comparators

The treatment group was treated with TCM alone or integrated traditional Chinese and Western medicine, while the control group was treated with conventional western medicine or placebo or waiting for treatment. TCM treatment mainly selects the relevant dosage forms of TCM, not limited to decoction, pill, injection, etc.

#### 2.3.3 Type of outcomes

##### 2.3.3.1 Primary outcomes

- Distant vision;
- Intraocular pressure;
- Diopter;

##### 2.3.3.2 Secondary outcomes

- Interpupillary distance and axis of eyeball;
- Adverse reaction.

#### 2.3.4 Study design

This study is a systematic review with NMA of randomized controlled trials (RCTs) on TCM decoction for the adolescent myopia. All relevant RCTs using TCM decoction for the adolescent myopia will be included. Quasi-RCTs will be excluded such as those allocating by medical record number. The specific participants, interventions, comparators, and outcomes criteria are as follows.

### 2.4 Literature retrieval strategy

Computer retrieval of published RCTs of TCM decoction for the adolescent myopia is conducted in PubMed, the Cochrane Library (issue 1, 2022), EMbase, China National Knowledge Infrastructure, China Biological Medicine, Chinese Scientific Journals Database (VIP), and wan-fang databases. The time limit of document retrieval is from the establishment of each database to January 31, 2022. The language is limited to English and Chinese. In addition, inclusive literature from the field and references from previous evaluations will be manually retrieved to find other potentially relevant articles. Search terms mainly include: “Myopia,” “Nearsightedness,” “Adolescent myopia,” “Traditional Chinese Medicine,” “Injection,” “Pill,” “Decoction,” “Powder,” “Eye ointment,” etc. Taking PubMed as an example, the initial retrieval strategy is shown in Table 1 and will be adjusted according to the specific database.

**Table 1:** Search strategy of the PubMed.

Number	Search terms
#1	Myopia[Mesh]
#2	Myopia[Title/Abstract] OR Nearsightedness[Title/Abstract]
#3	#1 OR #2
#4	Adolescent[Title/Abstract]
#5	Traditional Chinese medicine[Title/Abstract]
#6	Injection[Title/Abstract] OR Pill[Title/Abstract] OR Decoction[Title/Abstract] OR Powder[Title/Abstract] OR Eye ointment[Title/Abstract]
#7	#5 OR #6
#8	randomized controlled trial[Publication Type]
#9	controlled clinical trial[Publication Type]
#10	randomized[Title/Abstract]
#11	randomly[Title/Abstract]
#12	#8 OR #9 OR #10 OR #11
#13	#3 AND #4 AND #7 AND #12

### 2.5 Literature selection and data extraction

**Figure 1:**
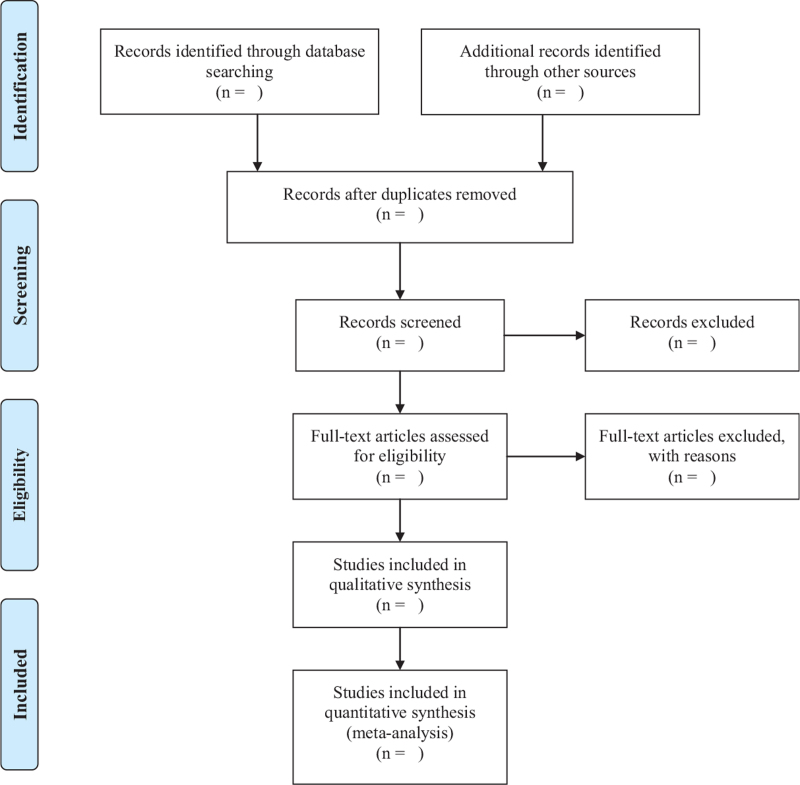
PRISMA flow diagram of the study selection process. PRISMA = Preferred Reporting Items for Systematic Reviews and Meta-Analysis.

As shown in Figure 1, Xiurong T and Zhongli S will independently screen literatures according to inclusion and exclusion criteria: (a) After importing the retrieved literature into EndNote X9.0, the duplicate literature was eliminated; (b) Conduct a preliminary screening by reading the headline summary to exclude literature that does not meet the inclusion criteria; (c) Reading the full text and making final selections; (d) Data extraction using a pre-designed data extraction table for the included literature and cross-checking the results; (e) In case of disagreement, the third researcher Penglong Y will be called upon to assist in judgment. Data extraction mainly included basic information of the literature (first author name, year of publication), basic information of study subjects (gender, average age, sample size, information of intervention and control group, intervention time, outcomes and follow-up time). At the same time, the key factors of bias risk assessment are extracted. We will contact the corresponding authors for additional information if necessary.

### 2.6 Quality assessment/methodological quality of included studies

Methodological quality will be assessed based on the bias tool (ROB) in Cochrane Handbook 5.1.0. Two trained researchers Yonghua L and Xianglin J will independently evaluate the risk of bias of the included studies. In case of dispute, submit to the third researcher Xingying L for arbitration. Cochrane bias risk assessment tool will be used to assess the risk of RCTs being included in NMA, 7 items are included^[16]^:

(a) random sequence generation, allocation concealment possibility;
(b) blinding of participants and personnel;
(c) blinding of outcome assessment;
(d) incomplete outcome data;
(e) selective reporting;
(f) other bias.

Based on the above 7 items, the included studies will be classified into three grades: low risk of bias, high risk of bias, and unclear risk of bias.

### 2.7 Data synthesis and statistical methods

#### 2.7.1 Network meta-analysis

This study uses Aggregate Data Drug Information System 1.16.8 for NMA, and uses Markov Chain-Monte Carlo algorithm to make Bayesian inference.^[17]^ Iteration operations were performed according to the following preset model parameters: 4 chains were used for simulation analysis, with initial value of 2.5, a step size of 10, annealing times of 20,000, and 50,000 simulation iteration times. Aggregate Data Drug Information System software is used to draw network evidence diagrams of different outcome indicators, and odds ratio or standardized mean differences is used for statistical analysis, both with 95% credible intervals. According to the results of the NMA, rank probability plot of various TCM decoction is generated and sorted by dominance, with Rank1 being the optimal sort.

#### 2.7.2 Statistical model selection

In this study, node-split model was used to analyze the consistency of data. When the statistical difference was compared directly and indirectly (*P* > .5), the consistency model was used for analysis. On the contrary, inconsistent model is adopted for analysis. If the consistency model is adopted, then the stability of the results is verified by the inconsistency model: when the inconsistency factors including 0, at the same time inconsistency standard deviation including 1 says the result of inconsistency model is more stable and reliable. At the same time, the pre-set parameters are used for iterative operation, and the convergence degree of iteration is judged by the potential scale reduced factor (PSRF). When the PSRF value is close to or equal to 1 (1 ≤ PSRF ≤ 1.05), the convergence is complete, indicating good stability of the model and reliable analysis results. If the PSRF is not in this range (1 ≤ PSRF ≤ 1.05), the iteration will continue manually until the PSRF value reaches the range standard.

#### 2.7.3 Heterogeneity test

Before the combination of effect size, the heterogeneity of the included literature is tested using STATA 15.0 software. Evaluate the heterogeneity between studies through *I*^2^. When *I*^2^ > 50%, it indicates that the heterogeneity between studies is large, using a random effect model; when *I*^2^ < 50%, it indicates that the heterogeneity between studies is small or there is no qualitative difference, using a fixed effect model. When the heterogeneity is greater, the source of heterogeneity should be further sought.

#### 2.7.4 Sensitivity analysis

If necessary, sensitivity analysis will be used to assess the impact of the studies on the random effects model. After each study was excluded one by one, the data analysis was carried out again to determine the stability of the results. If there is no qualitative change in the combined effect showed in the results, the results are stable.

#### 2.7.5 Subgroup analysis

If there is clinical and methodological heterogeneity, we will conduct a subgroup analysis of the patient's age, the degree of myopia, duration of treatment, or study quality.

#### 2.7.6 Publication bias

If 10 or more studies are included in the NMA, a comparison-adjusted funnel plot is developed using Stata to evaluate the presence of small sample effects or publication bias in the intervention network. If the plot is asymmetric and there is no inverted funnel shape, it indicates that there may be publication bias. The reasons may be related to the small sample size, allocation concealment, and insufficient implementation of blind method.

#### 2.7.7 Dealing with missing data

If the literature information is clearly incorrect or incomplete, we will contact the first author or the first author of the literature via email address. If no response is received, the document should be deleted.

#### 2.7.8 Evaluating the quality of the evidence

To grade evidence quality and understand the current situation of evidence rating thereby analyzing possible problems, The Grading of Recommendations Assessment, Development and Evaluation instrument will be used to assess the quality of evidence in the NMA.^[18]^ Based on bias, inconsistent, inaccurate, indirect, and the risk of publication bias (5 degradation factors), the quality classification for the four level of evidence: high, medium, low, and very low.

#### 2.7.9 Patient and public involvement

There was no patient or public involvement in the preparation of this protocol.

## 3 Discussions

Myopia has gradually become one of the leading eye diseases in the world. Studies have shown that TCM treatment with TCM decoction can effectively slow down the occurrence and development of myopia. Therefore, we hope to understand the efficacy and safety of various TCM decoction in the treatment of adolescent myopia through this study. Due to the diversity of clinical medication and to avoid large heterogeneity in the study, we could not include all categories of TCM decoction. There is a potential risk of bias. We will further optimize and deepen the research scheme according to actual needs. We will promptly disclose the reasons and timing of any changes that may be made.

## Author contributions

All the authors have approved the publication of the protocol.

**Conceptualization:** Xiurong Tian, Zhongli Sun, Penglong Yu.

**Data curation:** Xiurong Tian, Zhongli Sun, Yonghua Li.

**Formal analysis:** Xiurong Tian, Zhongli Sun.

**Funding acquisition:** Yonghua Li.

**Methodology:** Yonghua Li, Xianglin Jiang, Xingying Li.

**Project administration:** Xiurong Tian, Zhongli Sun, Yonghua Li.

**Writing – original draft:** Xiurong Tian, Zhongli Sun.

**Writing – review & editing:** Xiurong Tian, Zhongli Sun, Yonghua Li, Xianglin Jiang, Xingying Li, Penglong Yu.
